# The Effects of Epigallocatechin Gallate (EGCG) on Pulmonary Fibroblasts of Idiopathic Pulmonary Fibrosis (IPF)—A Next-Generation Sequencing and Bioinformatic Approach

**DOI:** 10.3390/ijms20081958

**Published:** 2019-04-22

**Authors:** Ming-Ju Tsai, Wei-An Chang, Ssu-Hui Liao, Kuo-Feng Chang, Chau-Chyun Sheu, Po-Lin Kuo

**Affiliations:** 1 Graduate Institute of Clinical Medicine, College of Medicine, Kaohsiung Medical University, Kaohsiung 807, Taiwan; SiegfriedTsai@gmail.com (M.-J.T.); 960215kmuh@gmail.com (W.-A.C.); s0970215575@gmail.com (S.-H.L.); kuopolin@seed.net.tw (P.-L.K.); 2 Division of Pulmonary and Critical Care Medicine, Department of Internal Medicine, Kaohsiung Medical University Hospital, Kaohsiung Medical University, Kaohsiung 807, Taiwan; 3 Department of Internal Medicine, School of Medicine, College of Medicine, Kaohsiung Medical University, Kaohsiung 807, Taiwan; 4 Department of Respiratory Therapy, School of Medicine, College of Medicine, Kaohsiung Medical University, Kaohsiung 807, Taiwan; 5 Welgene Biotech, Inc., Taipei 115, Taiwan; nickchang@welgene.com.tw

**Keywords:** bioinformatics, fibroblast, next-generation sequencing, epigallocatechin gallate, EGCG, *PCSK9*

## Abstract

Idiopathic pulmonary fibrosis (IPF) is a disabling and lethal chronic progressive pulmonary disease. Epigallocatechin gallate (EGCG) is a polyphenol, which is the major biological component of green tea. The anti-oxidative, anti-inflammatory, and anti-fibrotic effects of EGCG have been shown in some studies, whereas its effects in altering gene expression in pulmonary fibroblasts have not been systematically investigated. This study aimed to explore the effect of EGCG on gene expression profiles in fibroblasts of IPF. The pulmonary fibroblasts from an IPF patient were treated with either EGCG or water, and the expression profiles of mRNAs and microRNAs were determined by next-generation sequencing (NGS) and analyzed with the bioinformatics approach. A total of 61 differentially expressed genes and 56 differentially expressed microRNAs were found in EGCG-treated IPF fibroblasts. Gene ontology analyses revealed that the differentially expressed genes were mainly involved in the biosynthetic and metabolic processes of cholesterol. In addition, five potential altered microRNA–mRNA interactions were found, including hsa-miR-939-5p–*PLXNA4*, hsa-miR-3918–*CTIF*, hsa-miR-4768-5p–*PDE5A*, hsa-miR-1273g-3p–*VPS53*, and hsa-miR-1972–*PCSK9.* In summary, differentially expressed genes and microRNAs in response to EGCG treatment in IPF fibroblasts were identified in the current study. Our findings provide a scientific basis to evaluate the potential benefits of EGCG in IPF treatment, and warrant future studies to understand the role of molecular pathways underlying cholesterol homeostasis in the pathogenesis of IPF.

## 1. Introduction

Idiopathic pulmonary fibrosis (IPF) is a chronic progressive pulmonary disease characterized by progressive fibrosing interstitial pneumonitis [1,2,3,4,5]. Patients usually present with non-specific symptoms, such as exertional dyspnea and dry cough, and high-pitched fine inspiratory crackles (the so-called Velcro-like crackles) are usually heard in bilateral basal lung fields on auscultation [2]. A high-resolution computed tomography is a key diagnostic tool, which may reveal the pattern of usual interstitial pneumonia (UIP), characterized by “honey-combing” (subpleural multi-layer cystic lesions), traction bronchiectasis, and peripheral alveolar septal thickening, initially involving the basal and peripheral lungs with gradual progression to involve the whole lungs [3]. With increasing awareness in recent decades, incidence has risen over time, and estimated to be 2–30 cases per 100,000 person-years [2,3,6]. IPF has an increasing global burden, affecting about 3 million people worldwide [3]. Causing progressively disabling dyspnea, IPF has a devastating effect on patients’ quality of life and is a quite lethal disease [4]. The median survival of untreated IPF patients is around 3–5 years, which is worse than the majority of cancers in subjects with similar demographic characters [3,4,7].

Although the precise pathogenic mechanisms of IPF remain largely unclear, it is believed that fibroblasts play a key role in the development and progression of IPF [1,5,8]. Exaggerated proliferation, migration, and activation of fibroblasts, as well as their resistance to apoptosis, differentiation to myofibroblasts, and secretion of extracellular matrix components, have been proposed as a major pathogenic model of IPF [1,3,9,10,11]. Inflammation and oxidative stress are also commonly considered as major factors promoting pulmonary fibrosis [12,13,14].

The treatment modalities for IPF are currently limited. Only two medications, including pirfenidone, an anti-fibrotic agent, and nintedanib, a multi-target tyrosine kinase inhibitor, have been shown to be effective in reducing disease progression and improving quality of life [5,15,16,17,18,19,20,21]. Although these drugs offer hope for IPF patients, not all patients respond to these pharmacotherapies, and no biomarkers are available to predict the treatment response to any drug; therefore, efforts are continuously made to explore potential novel treatment modalities for IPF.

Epigallocatechin gallate (EGCG) is the ester from epigallocatechin and gallic acid [22]. This catechin is the major polyphenol responsible for the biological effects in tea [22,23]. In contrast to black tea, in which the flavan-3-ols are converted to theaflavins and thearubigins during fermentation processing, green tea retains the highest level of EGCG (100 g of dried leaves contain about 7380 mg of EGCG) [22,23]. EGCG is also found in trace amounts in some food sources, such as onions, hazelnuts, plums, and so on [24]. EGCG exhibits a wide range of therapeutic properties, such as anti-oxidative [25], anti-inflammatory [26], and anti-fibrotic effects [27]. It may modulate cell signaling pathways, such as mitogen-activated protein kinase (MAPK), NF-κB, and AMP-activated protein kinase (AMPK) pathways, and may also modulate epigenetic changes, such as DNA methylation and histone acetylation [22,23]. As a well-known mitochondrion-targeting medicinal agent, EGCG may also regulate mitochondrial metabolism, such as mitochondrial biogenesis and mitochondrial bioenergetics, as well as regulate cell cycle and apoptosis via mitochondria-mediated pathways [28].

Some in vitro and in vivo studies have shown the effects of EGCG on fibroblasts, such as attenuating cell proliferation, enhancing antioxidant defense systems, and inhibiting inflammation [27,29,30,31,32]. However, the effects of EGCG in modulating gene expression in pulmonary fibroblasts have not been systematically investigated. We therefore conducted this study to explore the effect of EGCG on gene expression profiles in fibroblasts of IPF using next-generation sequencing (NGS) and bioinformatic analyses.

## 2. Results

### 2.1. Gene Expression Profiling and Microrna Changes in IPF Fibroblasts Treated with EGCG

**Figure 1:**
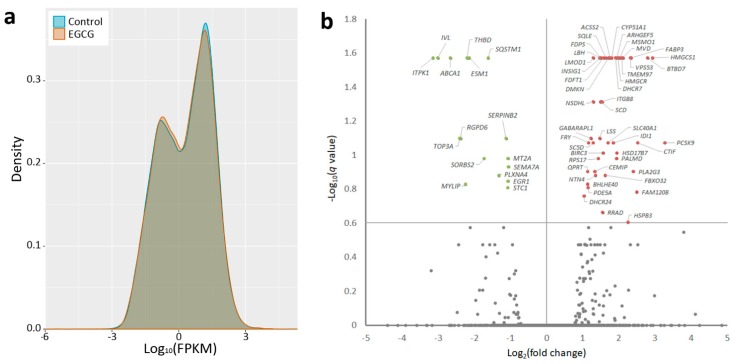
Differential gene expression patterns between idiopathic pulmonary fibrosis (IPF) fibroblasts treated with epigallocatechin gallate (EGCG) or water (control). (**a**) The frequency distribution of fragments per kilobase of transcript per million mapped reads (FPKM) between fibroblasts treated with EGCG or water (control) was compared and are presented in the density plot. (**b**) The volcano plot of –log_10_ (*q*-value) versus log_2_ (fold change) showed differentially downregulated (left upper quadrant) and upregulated (right upper quadrant) genes expressed in EGCG-treated IPF fibroblasts versus water-treated IPF fibroblasts. The genes with *q*-values >0.25 and >2-fold changes are plotted in green (downregulated) or red (upregulated).

The primary pulmonary fibroblasts from a patient with IPF were treated with EGCG or water, and RNAs were extracted from the cells and sent for NGS followed by bioinformatic analyses. Figure 1a shows the volcano plot of differentially expressed genes in EGCG-treated versus control fibroblasts. Genes with *q*-value <0.25 and >2-fold changes were selected for further analyses, including 16 significantly downregulated and 45 significantly upregulated genes in EGCG-treated versus control fibroblasts (Figure 1, Table A1). The small RNA-sequencing data generated with NGS were analyzed to identify potentially significant changes in microRNA profiles in EGCG-treated versus control fibroblasts. As shown in Table A2, we identified 56 microRNAs with >2-fold changes (22 upregulated and 34 downregulated).

### 2.2. Discovering the Altered microRNA–mRNA Interactions in IPF Fibroblasts Treated with EGCG

**Figure 2:**
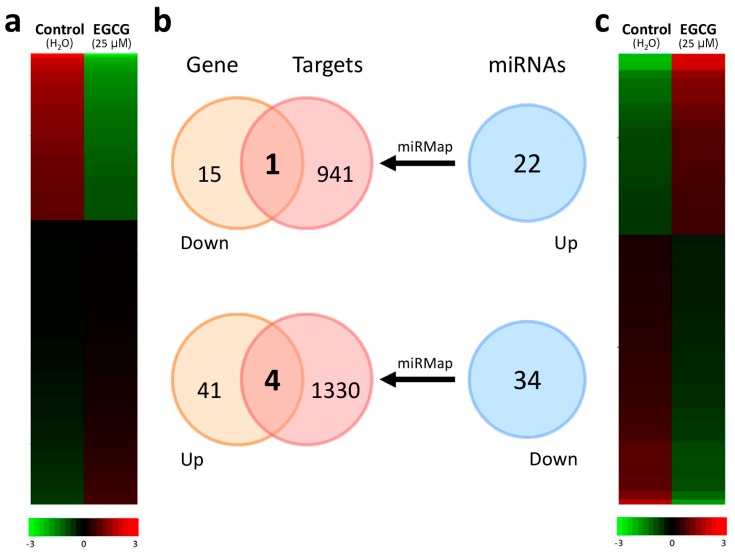
Differentially expressed genes and microRNAs with potential microRNA–target gene interactions identified in idiopathic pulmonary fibrosis (IPF) fibroblasts treated with epigallocatechin gallate (EGCG). A total of (**a**) 61 differentially expressed genes and (**c**) 56 differentially expressed microRNAs were identified in the EGCG-treated IPF fibroblasts with next-generation sequencing methods, and the heatmaps according to z-scores are illustrated. (**b**) Using the miRmap database for microRNA target prediction (selection criteria of miRmap score ≥97.0), 942 putative targets of the 22 upregulated microRNAs and 1334 putative targets of the 34 downregulated microRNAs were identified. Matching to the 16 downregulated genes and 45 upregulated genes identified in the EGCG-treated IPF fibroblasts, the intersection Venn diagram identified five potential microRNA–mRNA interactions (as shown in Table 1).

In order to discover altered microRNA–mRNA interactions in IPF fibroblasts treated with EGCG, we searched the putative targets of the microRNAs with >2-fold changes from NGS results using the miRmap database and selected those with miRmap scores >97.0. We matched the genes showing >2-fold changes with these putative targets. As shown in the intersection Venn diagram (Figure 2), five potential altered microRNA–mRNA interactions were found, including hsa-miR-939-5p–*PLXNA4*, hsa-miR-3918–*CTIF*, hsa-miR-4768-5p–*PDE5A*, hsa-miR-1273g-3p–*VPS53*, and hsa-miR-1972–*PCSK9* (Table 1). For further validation, we also searched these potential microRNA–mRNA interactions in various microRNA target predicting databases via miRWalk 2.0 [33], which included miRWalk, MicroT4, miRanda, miRDB, miRmap, RNA22, RNAhybrid, and TargetScan. Based on the criteria “microRNA target predicted in at least 6 (out of 8) databases”, all five potential altered microRNA–mRNA interactions were validated (Table 1).

### 2.3. Gene Ontology Annotations of the Differentially Expressed Genes in IPF Fibroblasts Treated with EGCG

Gene ontology analysis of the 61 differentially expressed genes was performed using DAVID, including cellular components, biological processes, and molecular functions. The cellular components significantly associated with these genes included endoplasmic reticulum (15 genes) and endoplasmic reticulum membrane (14 genes) (Table 2). The biological processes significantly associated with these genes included cholesterol biosynthetic process (15 genes), isoprenoid biosynthetic process (6 genes), oxidation-reduction process (11 genes), cholesterol biosynthetic process via lathosterol (3 genes), cholesterol biosynthetic process via desmosterol (3 genes), cholesterol homeostasis (5 genes), and steroid biosynthetic process (4 genes) (Table 2). The analyses failed to identify any molecular functions significantly associated with these genes. In the genes involved in these significantly associated cellular components and biological processes, *PCSK9*, which is significantly involved in cholesterol homeostasis, was the only gene targeted by a differentially expressed microRNA, hsa-miR-1972 (Table 2).

The KEGG pathways of the differentially expressed genes in EGCG-treated IPF fibroblasts included steroid biosynthesis (fold enrichment of 90.51), biosynthesis of antibiotics (fold enrichment of 11.95), terpenoid backbone biosynthesis (fold enrichment of 41.14), and metabolic pathways (fold enrichment of 2.82) (Table 3).

**Figure 3:**
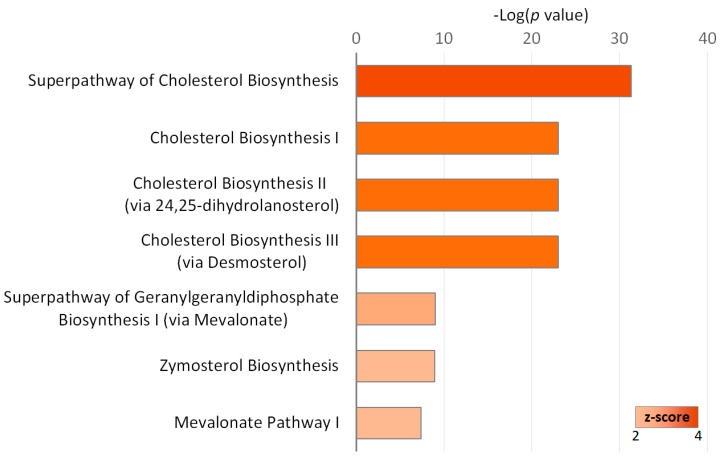
Canonical pathways significantly associated with the differentially expressed genes in idiopathic pulmonary fibrosis (IPF) fibroblasts treated with epigallocatechin gallate (EGCG) versus water. The z-score represents the magnitude of significant activation.

Using IPA, the canonical pathways associated with the 61 differentially expressed genes were investigated. As shown in Figure 3, we found that the associated canonical pathways included superpathway of cholesterol biosynthesis (15 genes), cholesterol biosynthesis I (10 genes), cholesterol biosynthesis II (via 24,25-dihydrolanosterol) (10 genes), cholesterol biosynthesis III (via desmosterol) (10 genes), superpathway of geranylgeranyl-diphosphate biosynthesis I (via mevalonate) (5 genes), zymosterol biosynthesis (4 genes), and mevalonate pathway I (4 genes).

**Figure 4:**
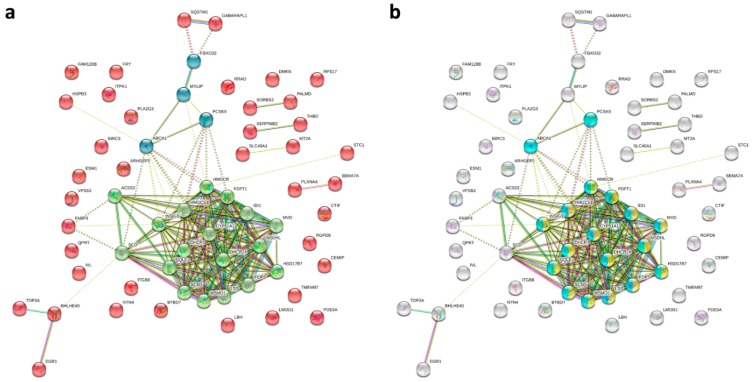
Protein–protein interaction (PPI) network analysis of the differentially expressed genes in idiopathic pulmonary fibrosis (IPF) fibroblasts treated with epigallocatechin gallate (EGCG) versus water using the Search Tool for the Retrieval of Interacting Genes (STRING) database. The 61 differentially expressed genes were input into the STRING database for PPI network analysis, and achieved a PPI network of 61 nodes and 172 edges, with PPI enrichment *p*-value <1.0 × 10^−16^. (**a**) Using k-means clustering, the network could be further clustered into three clusters (represented in green, blue, and red colors). (**b**) The genes associated with cholesterol biosynthetic process and cholesterol metabolic process are highlighted with yellow and light blue colors, respectively.

The protein–protein interaction (PPI) network analysis using the STRING database identified a total of 61 nodes and 172 edges, with PPI enrichment *p*-value <1.0 × 10^−16^. Using k-means clustering, the network could be further clustered into three clusters (Figure 4a). Within these three clusters, we found a core cluster, in which almost all genes were associated with cholesterol biosynthetic and metabolic processes (Figure 4b).

## 3. Discussion

The underlying mechanisms of IPF pathogenesis are quite complex, which might include oxidative stress, inflammation, enhanced proliferation and migration of fibroblasts, and so on [2,3,5,7,21]. As EGCG has anti-oxidative, anti-inflammatory, and anti-fibrotic effects, the current study tried to investigate the effects of EGCG on gene expression profiles in IPF fibroblasts. We found 61 differentially expressed genes in EGCG-treated IPF fibroblasts as compared with the control cells. Gene ontology analyses and network analyses revealed that these genes were mainly involved in the biosynthesis and metabolism of cholesterol. In addition, we also identified 56 differentially expressed microRNAs, and further analyses found that five potential altered microRNA–mRNA interactions, including hsa-miR-939-5p–*PLXNA4*, hsa-miR-3918–*CTIF*, hsa-miR-4768-5p–*PDE5A*, hsa-miR-1273g-3p–*VPS53*, and hsa-miR-1972–*PCSK9*, might be important changes in response to EGCG treatment in IPF fibroblasts. Among the five genes with potential microRNA–mRNA interactions, *PCSK9*, which might be upregulated in association with downregulated has-miR-1972, was the only one involved in cholesterol metabolism.

The effects and mechanisms of EGCG have been studied for various diseases. EGCG is considered beneficial for cardiovascular health and may prevent metabolic syndrome due to its effects in preventing or improving atherosclerosis, cardiac hypertrophy, myocardial infarction, diabetes, inflammation, and oxidative stress [34,35]. In type 2 diabetes and obesity, EGCG may modulate muscle homeostasis by increasing the expression of anti-oxidative enzymes, reversing the increased production of reactive oxygen species in skeletal muscle, regulating mitochondria-involved autophagy, stimulating glucose uptake, and increasing lipid oxidation [22]. As EGCG may also block the activity of peroxisome proliferator-activated receptors gamma (PPARγ) via binding to its active site, this polyphenol has been considered a potential anti-obesity compound [36]. Potential neuroprotective effects of EGCG have also been reported [37]. EGCG exhibits multiple immune-modulating effects, regulating intestinal mucosal immune responses, allergic diseases, as well as anticancer immunity [38]. In addition to its effects in inhibiting NF-κB, epithelial-mesenchymal transition, and cell invasion, the anticancer effects of EGCG might also involve the regulation of microRNAs and epigenetic mechanisms responsible for carcinogenesis and cancer progression [39,40].

A few previous studies have investigated the effect of EGCG on pulmonary fibroblasts. EGCG has been shown to inhibit the production of TNF-α, which might play an important role in the pathogenesis of IPF [41]. Sriram et al. have done a series of studies on the effects of EGCG in pulmonary fibrosis using the rat model of pulmonary fibrosis induced by intra-tracheal instillation of bleomycin [27,29,30,31]. EGCG augmented antioxidant activities and alleviated the bleomycin-induced oxidative stress, inflammation (increased levels of NF-κB, TNF-α, and IL-1β), and alveolar damage [30,31]. The increased glycoconjugates, increased activities of matrix degrading lysosomal enzymes, and ultrastructural changes induced by bleomycin were also attenuated by EGCG, suggesting the potential of EGCG as an anti-fibrotic agent [27]. EGCG also reversed the increased expression of gelatinases, including matrix metalloproteinase (MMP)-2 and MMP-9, TGF-β1, SMADs, and α-smooth muscle actin (α-SMA) induced by bleomycin treatment [29]. The in vitro studies using a fibroblast cell line also revealed that EGCG was capable of reversing the TGF-β1-induced proliferation and activation of fibroblasts [29]. Using a rat model of irradiation-induced pulmonary fibrosis, You et al. revealed that EGCG inhibited irradiation-induced alveolitis and pulmonary fibrosis through the effects such as inhibiting fibroblast proliferation, reducing collagen deposition, and regulating inflammatory cytokines [32].

The roles of cholesterol and lipoproteins in pulmonary diseases have been recognized in a few studies, although the detailed mechanisms remain unclear. Dyslipidemia affects innate and adaptive immunities in the lung [42]. Statins, 3-hydroxy-3-methylglutaryl-coenzyme A reductase (HMGCR) inhibitors originally designed as a cholesterol-lowering drug, also have HMGCR-independent properties, such as anti-inflammatory and anti-fibrotic effects. Pitavastatin and simvastatin inhibited TGF-β1-induced production of growth factors and fibrogenic mediators from lung fibroblasts [43,44]. In a mouse study, pravastatin attenuated bleomycin-induced pulmonary fibrosis by reducing the expression of inflammatory and growth factors, such as TGF-β1 and connective tissue growth factor, and the oxidative stress [45]. A large cross-sectional clinical study showed that lower high-density lipoprotein (HDL)-cholesterol was associated with more subclinical interstitial lung disease, shown by more high attenuation areas measured with computed tomography, and more extracellular matrix remodeling, shown by increased serum MMP-7 and surfactant protein-A [46]. Through gene ontology and functional analyses, we found that the EGCG-induced differentially expressed genes mainly involve the biosynthesis and metabolism of cholesterol. Further studies are needed to understand the roles of cholesterol-associated pathways in pulmonary fibrosis.

In this study, five differentially expressed genes were found as the potential targets of corresponding differentially expressed microRNAs, including downregulated *PLXNA4* and upregulated *CTIF*, *PDE5A*, *VPS53*, and *PCSK9*. As a study using the rat model of bleomycin-induced pulmonary fibrosis showed increased expression of *PLXNA4* [47], the downregulation of *PLXNA4* induced by EGCG might have beneficial effect in treating pulmonary fibrosis. In contrast to our findings that EGCG upregulated *PDE5A* expression in IPF fibroblasts, PDE5A inhibition by sildenafil improved bleomycin-induced pulmonary fibrosis by reducing oxidative stress [48]. *PCSK9* encodes proprotein convertase subtilisin/kexin type 9, which is a regulator of the homeostasis of plasma low-density lipoprotein (LDL)-cholesterol, and is associated with the metabolism of lipid and glucose [49]. Expression of *PCSK9* might reverse the abnormal cholesterol accumulation and the development of fibrosis in the liver caused by *E2F1* deficiency [50]. Although the roles of these genes in regulating the cell physiology of pulmonary fibroblasts remain largely unknown, these EGCG-induced gene expression alterations might provide potential targets to reverse pulmonary fibrosis and deserve further studies.

Some anti-fibrotic and pro-fibrotic microRNAs have been reported, and some of them might contribute to the pathogenesis of IPF [1,51,52]. The expression of miR-155 in human lung fibroblasts was upregulated by TNF-α and IL-1β and downregulated by TGF-β1; miR-155, which might target keratinocyte growth factor, promoted migration of fibroblasts and enhanced pulmonary fibrosis [53]. In studies using the mice model of bleomycin-induced pulmonary fibrosis, upregulation of miR-155 and downregulation of miR-29 were observed, which correlated with the degree of lung fibrosis [53,54]. The increased expression of miR-155 and decreased expression of miR-29 have been observed in the lungs of IPF patients [51]. In addition, greater expression and localization of miR-34a in pulmonary fibroblasts of IPF have been reported, which might function as an inhibiting mechanism of pulmonary fibrosis via inducing senescence and apoptosis of the fibroblasts [55]. Our findings that EGCG significantly upregulated miR-29b-2-5p and miR-34a-3p and downregulated miR-155-3p in IPF fibroblasts suggested a potential role of EGCG in the treatment of IPF through regulation of these microRNAs.

The dose of EGCG used in this study might be a concern. While most published in vitro studies used 10–100 μM of EGCG [56,57], we chose 25 μM of EGCG. As shown in a few previous studies, this dose of EGCG did not cause significant proliferation inhibition in human fibroblast cell line [29,58] and human colorectal cancer cell lines [59]. In line with these studies, our study showed that 25 μM of EGCG did not significantly alter the expression of genes related to cell proliferation or cell death. In addition, the bioavailability of EGCG is quite poor [56,60]. As shown in a pharmacokinetic study, the peak plasma EGCG level was only about 0.17 μM after drinking two cups of tea [56]. Parenteral routes, such as intravenous injection, might be needed to reach an effective systemic dose. However, a high systemic dose might cause hepatotoxicity [61]. In 2018, the European Food Safety Authority stated that taking ≥800 mg of EGCG daily might increase serum transaminase levels [62]. Further study is needed to determine the optimal systemic dose of EGCG. On the other hand, inhalation of an EGCG aerosol might possibly provide an alternative route of administration to increase EGCG concentration in the lungs while avoiding the potential systemic toxicity [63].

## 4. Materials and Methods

### 4.1. Cell Culture and Next-Generation Sequencing (NGS)

Human pulmonary fibroblasts from a patient of IPF (83-year-old Caucasian man, Catalog No. CC-7231), obtained from Lonza (Walkersville, MD, USA) were incubated at 37 °C in a 5% CO_2_-containing incubator in FGM™-2 Fibroblast Growth Medium-2 (Walkersville, MD, USA, Catalog No. CC-3132) containing 0.5 mL hFGF-B, 0.5 mL insulin, 10 mL FBS and 0.5 mL GA-1000. The medium was changed once every 2 or 3 days and the cells were channeled after distinct cell density. The cells were plated in 6-cm culture plates (1 × 10^5^ cells/well) and, after 24 h of incubation, treated with vehicle alone (ddH_2_O) or 25 μM of EGCG for 24 h. The dose of EGCG was chosen in reference to previous studies [29,56,57,58,59].

The mRNA and small RNA expression profiles were assessed using NGS as our previous studies [1,64,65,66,67,68]. In brief, total RNAs were extracted using TRIzol^®^ Reagent (Thermo Fisher Scientific, Waltham, MA, USA, Catalog No. 15596018) according to the instruction manual. The purified RNAs were quantified at OD_260nm_ using an ND-1000 spectrophotometer (NanoDrop Technologies, Wilmington, DE, USA) and qualitatively analyzed using a Bioanalyzer 2100 (Agilent Technologies, Santa Clara, CA, USA) with RNA 6000 LabChip kit (Agilent Technologies, Santa Clara, CA, USA). Library preparation and deep sequencing were carried out at Welgene Biotechnology Company (Taipei, Taiwan) as the official protocol of Illumina (San Diego, CA, USA).

For transcriptome sequencing, the library was constructed with Agilent’s SureSelect Strand Specific RNA Library Preparation Kit followed by AMPure XP Beads size selection. The sequence was directly determined using Illumina’s sequencing-by-synthesis (SBS) technology, and the sequencing data were generated by Welgene’s pipeline based on Illumina’s base-calling program bcl2fastq v2.2.0. For read alignment, HISAT2 [69], a fast and sensitive alignment program for mapping NGS reads to genomes, was used. The new indexing scheme of HISAT2 is based on the hierarchical graph Ferragina–Manzini index (GFM index) [70]. HISAT2 uses the global GFM index and a large set of small GFM indexes providing better performance for splicing junction alignment, which collectively covers the whole genome for rapid and accurate alignment, making HISAT2 an effective tool for transcriptome alignment. Differential expression analysis based on Cuffdiff (Cufflinks 2.2.1) [71] with genome bias detection/correction and Welgene in-house programs was performed. Differential expressed genes of each experiment design were followed by enrichment test for functional assay by clusterProfiler 3.6 [72]. The genes with low expression levels (<0.3 fragment per kilobase of transcript per million mapped reads (FPKM)) in both EGCG-treated and control fibroblasts and the genes with undetected level in either EGCG-treated or control fibroblasts were excluded. The *p*-values were calculated by Cuffdiff with non-grouped sample using “blind mode”, in which all samples were treated as replicates of a single global “condition” and used to build one model for statistical tests [71,73]. The *q*-values were *p*-value adjusted with false discovery rate using the method by Benjamini and Hochberg [74]. Genes with *q*-value < 0.25 (i.e., −log_10_ (*q*-value) > 0.602) and > 2-fold changes were considered significantly differentially expressed.

For small RNA sequencing, samples were prepared using Illumina sample preparation kit according to the TruSeq Small RNA Sample Preparation Guide. After the 3′ and 5′ adaptors were ligated to the RNA, reverse transcription followed by PCR amplification was performed. The enriched cDNA constructs were size-fractionated and purified on a 6% polyacrylamide gel electrophoresis and the bands containing the 18–40 nucleotide RNA fragments (140–155 nucleotides in length with both adapters) were extracted, which were then sequenced on an Illumina instrument (75 bp single-end reads). Sequencing data was processed with the Illumina software. After trimming and removing low-quality data with Trimmomatics v0.36 [75], the qualified data were analyzed with the miRDeep2 [76] to clip the 3′ adapter sequence and discard reads shorter than 18 nucleotides. The reads were then aligned to the human genome from the University of California, Santa Cruz (UCSC). Only reads that mapped perfectly to the genome ≤5 times were used for microRNA detection, because microRNAs usually map to few genomic locations. MiRDeep2 [76] estimates expression levels of known microRNAs, and also identifies novel microRNAs. The microRNAs with low levels (<1 normalized read per million (rpm)) in both EGCG-treated and control fibroblasts were excluded.

### 4.2. Analyses Using microRNA Target Predicting Databases

We used miRmap, an open-source software library providing comprehensive prediction of microRNA targets (http://mirmap.ezlab.org/) (accessed on 19 September 2018) [77], to predict the potential targets of significantly differentially expressed (>2-fold change) microRNAs. A miRmap score represents the repression strength of a microRNA on a target mRNA. Only the microRNA–mRNA pairs with miRmap score >97.0 were considered in the current study.

For further confirmation, we searched miRWalk 2.0 (http://zmf.umm.uni-heidelberg.de/apps/zmf/mirwalk2/) (accessed on 03 February 2019) [33], a comprehensive atlas of predicted and validated microRNA–target interactions, in which 12 computational target prediction databases were available. The information from eight databases, including miRWalk, MicroT4, miRanda, miRDB, miRmap, RNA22, RNAhybrid, and TargetScan, were extracted, because miRBridge, miRNAMap, PICTAR2, and PITA contained relatively little information. Putative microRNA targets suggested by at least six (out of eight) predicting databases were considered meaningful.

### 4.3. Database for Annotation, Visualization and Integrated Discovery (DAVID) Database Analysis

The DAVID (version 6.8) (https://david.ncifcrf.gov/) (accessed on 06 February 2019) [78] is a powerful tool for gene functional classification, which integrates many functional annotation databases, such as Gene Ontology (GO) biological process, and Kyoto Encyclopedia of Genes and Genomes (KEGG) pathway. By calculating the similarity of global annotation profiles with agglomeration algorithm method, a list of interesting genes can be classified into clusters based on their related biological functions, signaling pathways, or diseases. The differentially expressed genes were analyzed using the methods as in our previous studies [1,64,65,66,67,68]. The *p*-values (adjusted with false discovery rate using the method by Benjamini et al.) <0.05 were taken as the criteria of significance.

### 4.4. Ingenuity Pathway Analysis (IPA)

Ingenuity^®^ Pathway Analysis (IPA) software (Ingenuity Systems, Redwood City, CA, USA) integrates many research results and performs multiple analyses providing a comprehensive interpretation of big experimental data. We used IPA (version 2.3) to identify the canonical pathways associated with the candidate genes.

### 4.5. Search Tool for the Retrieval of Interacting Genes (STRING)

The STRING database (version 11.0) (https://string-db.org/) (accessed on 7 February 2019) covers 5090 organisms, 24.6 million proteins and >2000 million interactions that provides analysis and integration of direct and indirect protein–protein interactions (PPI), and focuses on functional association [79]. The differentially expressed genes identified were uploaded, and interactions with at least medium confidence (interaction score >0.4) were selected. Network was clustered using k-means clustering to a specified number of clusters.

## 5. Conclusions

In summary, our study demonstrated an innovative model to discover the potential effects of a nature compound on gene expression alterations and microRNA changes using an NGS and bioinformatic approach. We identified differentially expressed genes and microRNAs in response to EGCG treatment in IPF fibroblasts. These gene expression changes were mainly involved in the biosynthesis and metabolism of cholesterol, suggesting that EGCG might have effects on IPF treatment through regulation of the cholesterol-associated genes. Our findings provide a scientific basis to evaluate the potential benefits of EGCG in IPF treatment, and warrant future studies to understand the role of molecular pathways underlying cholesterol homeostasis in the pathogenesis of IPF.

## Figures and Tables

**Table 1:** Potential altered miRNA–mRNA interactions in idiopathic pulmonary fibrosis (IPF) fibroblasts treated with epigallocatechin gallate (EGCG).

Gene			miRNA		microRNA-Target Gene Prediction in Various Databases *							
Official Symbol	Gene Name	Log_2_ (ratio)	miRNA Name	Fold Change	mirmap Score	miRWalk	Microt4	miRanda	miRDB	RNA22	RNAhybrid	TargetScan
*PLXNA4*	plexin A4	−1.32	hsa-miR-939-5p	4.85	99.97	Yes	Yes	Yes	Yes	Yes	Yes	Yes
*CTIF*	cap binding complex dependent translation initiation factor	2.52	hsa-miR-3918	−4.19	99.97	Yes	Yes	Yes	Yes	Yes	Yes	Yes
*PDE5A*	phosphodiesterase 5A	1.14	hsa-miR-4768-5p	−2.75	99.34	Yes	Yes	Yes	No	No	Yes	Yes
*VPS53*	VPS53, GARP complex subunit	2.32	hsa-miR-1273g-3p	−2.03	99.76	Yes	Yes	Yes	No	No	Yes	Yes
*PCSK9*	proprotein convertase subtilisin/kexin type 9	3.27	hsa-miR-1972	−3.11	99.58	Yes	Yes	Yes	No	Yes	Yes	Yes

**Table 2:** Gene ontology analysis of the 61 differentially expressed genes in idiopathic pulmonary fibrosis (IPF) fibroblasts treated with epigallocatechin gallate (EGCG) using the Database for Annotation, Visualization and Integrated Discovery (DAVID).

Category and Term	Gene Count	Genes	Fold Enrichment	*p*-Value	Adjusted *p*-Value
**Cellular components**					
Endoplasmic reticulum	15	*GABARAPL1, MSMO1, HMGCR, CYP51A1, SCD, FDFT1, SQSTM1, SQLE, DHCR7, INSIG1, CEMIP, PCSK9, HSD17B7, NSDHL, DHCR24*	5.41	3.54 × 10^−7^	3.75 × 10^−5^
Endoplasmic reticulum membrane	14	*SC5D, MSMO1, CYP51A1, SQLE, HMGCR, SCD, DHCR7, INSIG1, LSS, ABCA1, HSD17B7, NSDHL, FDFT1, DHCR24*	4.85	3.55 × 10^−6^	1.88 × 10^−4^
**Biological processes**					
Cholesterol biosynthetic process	15	*MSMO1, MVD, HMGCR, CYP51A1, HMGCS1, FDPS, LSS, FDFT1, SQLE, DHCR7, INSIG1, IDI1, HSD17B7, NSDHL, DHCR24*	108.66	9.71 × 10^−26^	4.19 × 10^−23^
Isoprenoid biosynthetic process	6	*MVD, HMGCR, FDPS, HMGCS1, IDI1, FDFT1*	117.98	9.59 × 10^−10^	2.07 × 10^−7^
Oxidation-reduction process	11	*SC5D, MSMO1, SQLE, HMGCR, CYP51A1, SCD, DHCR7, HSD17B7, NSDHL, FDFT1, DHCR24*	5.11	4.23 × 10^−5^	0.0061
Cholesterol biosynthetic process via lathosterol	3	*SC5D, DHCR7, DHCR24*	206.46	7.50 × 10^−5^	0.0080
Cholesterol biosynthetic process via desmosterol	3	*SC5D, DHCR7, DHCR24*	206.46	7.50 × 10^−5^	0.0080
Cholesterol homeostasis	5	*TMEM97, FABP3, **PCSK9**, MYLIP, ABCA1*	21.51	7.97 × 10^−5^	0.0068
Steroid biosynthetic process	4	*CYP51A1, LSS, NSDHL, FDFT1*	34.41	0.0002	0.0143

* *p*-values adjusted with false discovery rate using the method by Benjamini et al. The gene predicted as the target of the significantly dysregulated microRNA (Table 1) is highlighted with bold font and underlined.

**Table 3:** Kyoto Encyclopedia of Genes and Genomes (KEGG) pathway analysis of the significantly differentially expressed genes in idiopathic pulmonary fibrosis (IPF) fibroblasts treated with epigallocatechin gallate (EGCG).

Description	Count	*p*-value	Adjusted *p*-value *	Genes	Fold Enrichment
Steroid biosynthesis	10	2.11 × 10^−16^	1.42 × 10^−14^	*SC5D*, *MSMO1*, *SQLE*, *CYP51A1*, *DHCR7*, *LSS*, *HSD17B7*, *NSDHL*, *FDFT1*, *DHCR24*	90.51
Biosynthesis of antibiotics	14	2.91 × 10^−11^	9.31 × 10^−10^	*SC5D*, *MSMO1*, *MVD*, *CYP51A1*, *SQLE*, *HMGCR*, *FDPS*, *HMGCS1*, *LSS*, *IDI1*, *ACSS2*, *HSD17B7*, *NSDHL*, *FDFT1*	11.95
Terpenoid backbone biosynthesis	5	4.84 × 10^−6^	1.03 × 10^−4^	*MVD*, *HMGCR*, *FDPS*, *HMGCS1*, *IDI1*	41.14
Metabolic pathways	19	1.53 × 10^−5^	2.45 × 10^−4^	*SC5D*, *MSMO1*, *MVD*, *CYP51A1*, *HMGCR*, *HMGCS1*, *FDPS*, *LSS*, *ACSS2*, *FDFT1*, *SQLE*, *DHCR7*, *QPRT*, *PLA2G3*, *ITPK1*, *IDI1*, *HSD17B7*, *NSDHL*, *DHCR24*	2.82

* *p*-values adjusted with false discovery rate using the method by Benjamini et al.

## Appendix A

**Table A1:** Differentially expressed genes in idiopathic pulmonary fibrosis (IPF) fibroblasts treated with epigallocatechin gallate (EGCG) versus water (control).

Official Gene Symbol	FPKM		Ratio (EGCG/Control)	Log_2_(ratio)	*p*-Value	*q*-Value *
	EGCG	Control				
*PCSK9*	0.85	0.09	9.65	3.27	0.0003	0.0846
*HMGCS1*	441.43	57.81	7.64	2.93	0.0001	0.0268
*BTBD7*	7.42	1.07	6.96	2.80	0.0001	0.0268
*CTIF*	5.12	0.89	5.75	2.52	0.0003	0.0846
*FAM120B*	2.44	0.43	5.64	2.50	0.0007	0.1650
*PLA2G3*	1.29	0.24	5.29	2.40	0.0005	0.1250
*FABP3*	117.28	23.12	5.07	2.34	0.0001	0.0268
*VPS53*	10.80	2.16	4.99	2.32	0.0001	0.0268
*HSPB3*	5.34	1.12	4.76	2.25	0.0011	0.2481
*MVD*	96.80	22.27	4.35	2.12	0.0001	0.0268
*TMEM97*	140.13	32.98	4.25	2.09	0.0001	0.0268
*MSMO1*	224.89	55.13	4.08	2.03	0.0001	0.0268
*HMGCR*	136.04	34.55	3.94	1.98	0.0001	0.0268
*ARHGEF5*	3.48	0.90	3.86	1.95	0.0001	0.0268
*HSD17B7*	11.98	3.12	3.85	1.94	0.0003	0.0973
*PALMD*	3.68	0.96	3.82	1.93	0.0004	0.1047
*DHCR7*	84.31	22.39	3.76	1.91	0.0001	0.0268
*IDI1*	185.79	51.74	3.59	1.84	0.0003	0.0846
*CYP51A1*	329.34	94.32	3.49	1.80	0.0001	0.0268
*ACSS2*	25.91	7.69	3.37	1.75	0.0001	0.0268
*DMKN*	2.32	0.71	3.28	1.71	0.0001	0.0268
*SLC40A1*	7.87	2.43	3.24	1.70	0.0003	0.0846
*SQLE*	108.28	34.77	3.11	1.64	0.0001	0.0268
*FBXO32*	5.71	1.85	3.08	1.62	0.0005	0.1319
*FDFT1*	206.04	69.04	2.98	1.58	0.0001	0.0268
*BIRC3*	12.03	4.07	2.95	1.56	0.0003	0.0973
*RRAD*	4.19	1.43	2.94	1.55	0.0010	0.2174
*ITGB8*	6.31	2.17	2.91	1.54	0.0001	0.0486
*FDPS*	260.06	91.77	2.83	1.50	0.0001	0.0268
*SCD*	377.97	134.01	2.82	1.50	0.0001	0.0486
*INSIG1*	268.58	95.40	2.82	1.49	0.0001	0.0268
*LSS*	129.93	46.81	2.78	1.47	0.0002	0.0798
*LBH*	128.19	46.39	2.76	1.47	0.0001	0.0268
*RPS17*	60.77	22.54	2.70	1.43	0.0004	0.1047
*NTN4*	12.06	4.72	2.56	1.35	0.0005	0.1319
*CEMIP*	216.43	85.86	2.52	1.33	0.0005	0.1250
*LMOD1*	15.53	6.33	2.46	1.30	0.0001	0.0268
*NSDHL*	53.96	22.04	2.45	1.29	0.0001	0.0486
*SC5D*	93.06	38.02	2.45	1.29	0.0003	0.0846
*GABARAPL1*	20.49	8.74	2.34	1.23	0.0002	0.0798
*FRY*	6.83	3.06	2.23	1.16	0.0003	0.0846
*PDE5A*	278.27	125.88	2.21	1.14	0.0007	0.1556
*BHLHE40*	16.48	7.50	2.20	1.13	0.0006	0.1482
*QPRT*	12.85	5.85	2.20	1.13	0.0005	0.1250
*DHCR24*	151.61	73.96	2.05	1.04	0.0008	0.1742
*SEMA7A*	26.51	54.97	0.48	-1.05	0.0004	0.1174
*MT2A*	352.79	738.65	0.48	-1.07	0.0004	0.1047
*EGR1*	17.36	36.40	0.48	-1.07	0.0006	0.1426
*STC1*	41.69	87.70	0.48	-1.07	0.0007	0.1556
*SERPINB2*	131.57	286.07	0.46	-1.12	0.0002	0.0798
*PLXNA4*	2.83	7.07	0.40	-1.32	0.0005	0.1319
*SQSTM1*	11.91	36.40	0.33	-1.61	0.0001	0.0268
*SORBS2*	0.12	0.41	0.30	-1.73	0.0004	0.1047
*ESM1*	10.28	45.34	0.23	-2.14	0.0001	0.0268
*THBD*	3.13	14.37	0.22	-2.20	0.0001	0.0268
*MYLIP*	0.61	2.91	0.21	-2.24	0.0006	0.1482
*RGPD6*	0.57	2.99	0.19	-2.38	0.0002	0.0798
*TOP3A*	0.42	2.21	0.19	-2.40	0.0002	0.0798
*ABCA1*	2.09	13.21	0.16	-2.66	0.0001	0.0268
*IVL*	0.13	1.03	0.12	-3.01	0.0001	0.0268
*ITPK1*	0.85	7.50	0.11	-3.14	0.0001	0.0268

* *p*-values adjusted with false discovery rate using the method by Benjamini et al. IPF, idiopathic pulmonary fibrosis; EGCG, epigallocatechin gallate; FPKM, fragments per kilobase of transcript per million mapped reads.

**Table A2:** MicroRNAs with significant change in idiopathic pulmonary fibrosis (IPF) fibroblasts treated with epigallocatechin gallate (EGCG) versus water (control).

miRNA	Precursor	Normalized Read Count (rpm)		Fold Change	Up/Down
		EGCG	Control		
hsa-miR-491-3p	hsa-mir-491	1.39	0.08	17.38	Up
hsa-miR-4803	hsa-mir-4803	1.21	0.08	15.13	Up
hsa-miR-1322	hsa-mir-1322	1.04	0.17	6.12	Up
hsa-miR-939-5p	hsa-mir-939	1.65	0.34	4.85	Up
hsa-miR-101-5p	hsa-mir-101-1	1.13	0.25	4.52	Up
hsa-miR-141-3p	hsa-mir-141	1.82	0.42	4.33	Up
hsa-miR-33a-3p	hsa-mir-33a	3.03	0.84	3.61	Up
hsa-miR-34a-3p	hsa-mir-34a	6.5	1.93	3.37	Up
hsa-miR-503-3p	hsa-mir-503	1.21	0.42	2.88	Up
hsa-miR-200c-3p	hsa-mir-200c	1.3	0.5	2.60	Up
hsa-miR-145-5p	hsa-mir-145	93.71	37.01	2.53	Up
hsa-miR-491-5p	hsa-mir-491	1.91	0.76	2.51	Up
hsa-miR-770-5p	hsa-mir-770	2.51	1.01	2.49	Up
hsa-miR-29b-2-5p	hsa-mir-29b-2	2.6	1.09	2.39	Up
hsa-miR-548at-5p	hsa-mir-548at	2.17	0.93	2.33	Up
hsa-miR-4636	hsa-mir-4636	2.51	1.09	2.30	Up
hsa-miR-3684	hsa-mir-3684	1.65	0.76	2.17	Up
hsa-miR-6723-5p	hsa-mir-6723	1.65	0.76	2.17	Up
hsa-miR-142-5p	hsa-mir-142	1.39	0.67	2.07	Up
hsa-miR-655-3p	hsa-mir-655	4.85	2.35	2.06	Up
hsa-miR-3130-5p	hsa-mir-3130-2	1.56	0.76	2.05	Up
hsa-miR-3613-3p	hsa-mir-3613	1.21	0.59	2.05	Up
hsa-miR-1273g-3p	hsa-mir-1273g	0.87	1.77	−2.03	Down
hsa-miR-2116-3p	hsa-mir-2116	2.51	5.13	−2.04	Down
hsa-miR-3622a-5p	hsa-mir-3622a	0.69	1.43	−2.07	Down
hsa-miR-5699-5p	hsa-mir-5699	1.65	3.45	−2.09	Down
hsa-miR-138-1-3p	hsa-mir-138-1	55.39	117.24	−2.12	Down
hsa-miR-340-3p	hsa-mir-340	3.81	8.24	−2.16	Down
hsa-miR-423-5p	hsa-mir-423	822.58	1779.79	−2.16	Down
hsa-miR-25-5p	hsa-mir-25	27.39	59.54	−2.17	Down
hsa-miR-3605-3p	hsa-mir-3605	12.05	26.66	−2.21	Down
hsa-miR-1910-5p	hsa-mir-1910	22.71	50.63	−2.23	Down
hsa-miR-4745-5p	hsa-mir-4745	0.95	2.19	−2.31	Down
hsa-miR-6840-5p	hsa-mir-6840	0.61	1.43	−2.34	Down
hsa-miR-92a-1-5p	hsa-mir-92a-1	8.06	19.09	−2.37	Down
hsa-miR-1914-5p	hsa-mir-1914	0.52	1.26	−2.42	Down
hsa-miR-937-3p	hsa-mir-937	4.33	10.85	−2.51	Down
hsa-miR-323a-5p	hsa-mir-323a	0.43	1.09	−2.53	Down
hsa-miR-3648	hsa-mir-3648-1	0.78	2.02	−2.59	Down
hsa-miR-3648	hsa-mir-3648-2	0.78	2.02	−2.59	Down
hsa-miR-1228-3p	hsa-mir-1228	0.52	1.35	−2.6	Down
hsa-miR-1294	hsa-mir-1294	0.69	1.85	−2.68	Down
hsa-miR-3177-5p	hsa-mir-3177	0.69	1.85	−2.68	Down
hsa-miR-4768-5p	hsa-mir-4768	0.52	1.43	−2.75	Down
hsa-miR-197-5p	hsa-mir-197	0.69	2.1	−3.04	Down
hsa-miR-1972	hsa-mir-1972-1	0.35	1.09	−3.11	Down
hsa-miR-1972	hsa-mir-1972-2	0.35	1.09	−3.11	Down
hsa-miR-3691-5p	hsa-mir-3691	1.13	3.7	−3.27	Down
hsa-miR-548al	hsa-mir-548al	0.35	1.18	−3.37	Down
hsa-miR-4766-3p	hsa-mir-4766	0.43	1.77	−4.12	Down
hsa-miR-3918	hsa-mir-3918	0.26	1.09	−4.19	Down
hsa-miR-548t-5p	hsa-mir-548t	0.26	1.09	−4.19	Down
hsa-miR-6783-3p	hsa-mir-6783	0.26	1.09	−4.19	Down
hsa-miR-155-3p	hsa-mir-155	0.26	1.18	−4.54	Down
hsa-miR-184	hsa-mir-184	0.61	2.78	−4.56	Down
hsa-miR-6859-5p	hsa-mir-6859-1	0.17	1.18	−6.94	Down
hsa-miR-6859-5p	hsa-mir-6859-2	0.17	1.18	−6.94	Down
hsa-miR-6859-5p	hsa-mir-6859-3	0.17	1.18	−6.94	Down
hsa-miR-6859-5p	hsa-mir-6859-4	0.17	1.18	−6.94	Down
hsa-miR-1304-5p	hsa-mir-1304	0.09	1.43	−15.89	Down
hsa-miR-891a-5p	hsa-mir-891a	0.09	1.43	−15.89	Down

IPF, idiopathic pulmonary fibrosis; EGCG, epigallocatechin gallate; rpm, reads per million.
